# Envejecimiento saludable en la Región de las Américas^*^

**DOI:** 10.26633/RPSP.2021.125

**Published:** 2021-09-13

**Authors:** Jarbas Barbosa da Silva, John W. Rowe, José Ricardo Jauregui

**Affiliations:** 1 Organización Panamericana de la Salud Washington, DC Estados Unidos de América Organización Panamericana de la Salud, Washington, DC, Estados Unidos de América; 2 Asociación Internacional de Gerontología y Geriatría y Escuela de Salud Pública Mailman, Universidad de Columbia Nueva York Estados Unidos de América Asociación Internacional de Gerontología y Geriatría y Escuela de Salud Pública Mailman, Universidad de Columbia, Nueva York, Estados Unidos de América; 3 Asociación Internacional de Gerontología y Geriatría Nueva York Estados Unidos de América Asociación Internacional de Gerontología y Geriatría, Nueva York, Estados Unidos de América

**Keywords:** Envejecimiento, envejecimiento saludable, América Latina, Región del Caribe, Américas

Tan solo en los últimos 50 años, la esperanza de vida ha aumentado en más de 20 años. Este aumento significativo de la longevidad se debe en parte a los avances de la medicina, las intervenciones de salud pública, la biotecnología y el desarrollo social y económico, que han permitido a las personas vivir más que en cualquier otro momento de la historia (1).

En América Latina y el Caribe, la proporción de personas de 60 o más años de edad aumentará incluso en 18% durante el próximo decenio y para el 2050 se ubicará entre 25% y 30% de la población. Esta transición ocurrirá en 35 años, que es tan solo la mitad del tiempo que requirió en Estados Unidos y Canadá (2). Debido a esta acelerada transición demográfica, la denominada “ventana de oportunidad demográfica”, que es el tiempo disponible para prepararse para la transición demográfica, se está reduciendo rápidamente en la Región de las Américas. Aunque cada vez se entiende más que es urgente dar prioridad al tema del envejecimiento, deberán realizarse mayores esfuerzos para abordar este cambio demográfico inminente. Son necesarias acciones e intervenciones específicas con objeto de asegurar que la longevidad y el envejecimiento sean resultados positivos del desarrollo sostenible en la Región (3). Los cambios demográficos, junto con las transiciones epidemiológicas y otros retos como la migración y el cambio climático, exigen a los países la creación de mecanismos innovadores para abordar estas nuevas realidades en todos los sectores (4).

A pesar de la predictibilidad de envejecimiento de la población, el mundo dista mucho de estar preparado para abordar esta transición demográfica. La pandemia de COVID-19 ha arrojado luz sobre muchas brechas existentes en lo que hacemos y en la manera en que pensamos acerca del envejecimiento y las personas mayores. La pandemia ha puesto de manifiesto la forma generalizada en la que se presenta el edadismo —estereotipos, prejuicios y discriminación a causa de la edad— en la sociedad, en particular en los sistemas de salud y las organizaciones que prestan servicios de salud. Muchas decisiones relativas a la atención de salud, el uso de recursos de los sistemas de salud y las medidas destinadas a contener la propagación del virus, como los debates sobre el aislamiento vertical, se han basado exclusivamente en la edad cronológica (5).

Las situaciones actuales observadas durante la pandemia de COVID-19, junto con la revolución generada por la longevidad en la Región, invitan a establecer una nueva visión para el futuro, y visualizar un mundo en el que las personas mayores sean miembros valiosos, activos y productivos de la sociedad (6). En este contexto y con el objetivo para mejorar la vida de las personas mayores, la Década del Envejecimiento Saludable (2021-2030) fue aprobada por la Asamblea Mundial de la Salud en agosto del 2020 y proclamada por la Asamblea General de las Naciones Unidas en diciembre del 2020. La Década del Envejecimiento Saludable es la principal estrategia para alcanzar y prestar apoyo a las acciones destinadas a construir una sociedad para todas las edades, con la perspectiva de promover un mundo en el cual todos puedan tener una vida larga y sana. Esta estrategia requiere una acción colectiva y concertada durante el próximo decenio por parte de los gobiernos, las organizaciones regionales e internacionales, la sociedad civil, el sector privado, la comunidad académica y los medios de comunicación, con objeto de conseguir que la edad avanzada sea realmente una oportunidad para todos (7).

La Década del Envejecimiento Saludable se centrará en cuatro áreas principales de acción que incluyen: cambiar la forma en la que pensamos, sentimos y actuamos respecto a la edad y el envejecimiento; asegurar que las comunidades fomenten las capacidades de las personas mayores; proporcionar una atención integrada y centrada en la persona y servicios de atención primaria de salud que respondan a las personas mayores; y brindar acceso a cuidados a largo plazo a las personas mayores que lo necesiten (8).

Se basa también en el aprovechamiento de cuatro elementos facilitadores que contribuirán al logro de los resultados en las cuatro áreas de acción principales. Uno de ellos es el fortalecimiento de los datos, la investigación y la innovación. Tres cuartas partes de los países del mundo no disponen de datos o tienen tan solo datos limitados sobre el envejecimiento saludable o sobre los grupos de personas mayores. En el 2020, en la Región de las Américas, tan solo 15 países afirmaron que cuentan con datos transversales y representativos a nivel nacional del estado de salud y de las necesidades de las personas mayores, y únicamente 10 indicaron que disponen de estudios longitudinales representativos de este tipo que sean de dominio público. Esta falta de investigación contribuye a producir la invisibilidad y la exclusión de las personas mayores. A fin de abordar a este grupo heterogéneo de una manera integral, deben desglosarse los datos para conocer mejor el estado de salud de las personas mayores, su bienestar económico y social, y sus conexiones en la comunidad (1). Ha habido una falta de puesta en común y presentación de los datos desglosados según la edad, incluso en situaciones en las que las personas mayores tienen una mayor vulnerabilidad, como las emergencias, los desastres y determinados trastornos crónicos. Por ejemplo, durante la pandemia de COVID-19, hubo una falta de datos desglosados según la edad en cuanto a los casos notificados o las muertes, especialmente en los países de ingresos bajos y medianos.

La investigación sobre el envejecimiento saludable debe abordar las brechas existentes y las necesidades actuales de las personas mayores, prever los retos para el futuro y vincular las condiciones sociales, biológicas, económicas y ambientales y los determinantes del envejecimiento saludable de la primera y la segunda mitad de la vida, así como elaborar intervenciones destinadas a mejorar las trayectorias del envejecimiento saludable (8). Adquirir más conocimientos sobre las personas mayores y el envejecimiento saludable por medio de investigaciones y crear una base científica sólida sobre esta materia ayudarán en la toma de decisiones fundamentadas y basadas en la evidencia, así como en la evaluación y promoción de intervenciones costoeficaces que generen mejoras importantes en la salud y el bienestar de las sociedades que están envejeciendo (3).

Con objeto de resaltar la importancia de la investigación sobre el envejecimiento saludable en la Región de las Américas y presentar investigaciones de interés generadas en la Región, la Organización Panamericana de la Salud (OPS) ha elaborado esta edición especial de la *Revista Panamericana de Salud Pública* sobre el envejecimiento saludable, centrándose en América Latina. En el contexto de la Década del Envejecimiento Saludable y reconociendo la importancia de la colaboración en los esfuerzos por fortalecer esta área, la Organización Panamericana de la Salud ha colaborado con la Asociación Internacional de Gerontología y Geriatría (IAGG) en la elaboración de esta edición especial, de acuerdo con su misión de promover los niveles más altos de logro en la investigación gerontológica, y de interactuar con otras organizaciones en la promoción de los intereses gerontológicos.
